# Combined miRNA transcriptome and proteome analysis of extracellular vesicles in urine and blood from the Pompe mouse model

**DOI:** 10.1080/07853890.2024.2402503

**Published:** 2024-10-24

**Authors:** David Merberg, Rodney Moreland, Zhenqiang Su, Bin Li, Bob Crooker, Kathleen Palmieri, Simon W. Moore, Andrew Melber, Ruby Boyanapalli, Galen Carey, Mahindra Makhija

**Affiliations:** a Takeda Pharmaceutical Company Limited, Rare Disease Drug Discovery Unit, Cambridge, MA, USA; b Takeda Pharmaceutical Company Limited, Preclinical and Translational Sciences, Cambridge, MA, USA; c Takeda International – UK, London, England

**Keywords:** Pompe disease, exosomes, biomarker, miRNA, proteomes

## Abstract

**Introduction:**

Acid α-glucosidase (GAA) is a lysosomal enzyme that hydrolyzes glycogen to glucose. Deficiency of GAA causes Pompe disease (PD), also known as glycogen storage disease type II. The resulting glycogen accumulation causes a spectrum of disease severity ranging from infantile-onset PD to adult-onset PD. Additional non-invasive biomarkers of disease severity are needed to monitor response to therapeutic interventions.

**Methods:**

We measured protein and miRNA abundance in exosomes from serum and urine from the PD mouse model (B6;129-GaaTm1Rabn/J), wild-type mice, and PD mice treated with a candidate gene therapy.

**Results:**

There were significant differences in the abundance of 113 miRNA in serum exosomes from Pompe versus healthy mice. Levels of miR-206, miR-133, miR-1a, miR-486, and other important regulators of muscle development and maintenance were altered in the Pompe samples. The serum and urine exosome proteomes of healthy and Pompe mice also differed broadly. Several of the dysregulated proteins are encoded by genes with potential target sites for affected miRNA.

**Conclusion:**

Exosomes derived from urine or serum are a potential source of biomarkers for Pompe Disease. Further study of the differences in the miRNA transcriptome and proteome content of exosomes may yield new insights into disease mechanisms.

## Introduction

Glycogen storage disease type II, also known as Pompe disease or acid maltase deficiency, is caused by a deficiency of acid α-glucosidase (GAA; EC 3.2.1.3), which hydrolyzes glycogen to glucose. Loss or reduction of GAA activity results in glycogen accumulation in lysosomes leading to damage in respiratory, cardiac, and skeletal muscle. Disease severity ranges from a rapidly progressing infantile course (Infantile Onset Pompe Disease, IOPD) to a slower progressing and extremely heterogeneous course (Late Onset Pompe Disease, LOPD). Patients with classic infantile onset typically show hypertrophic cardiomyopathy and die before 1 year of age if left untreated. These patients can be further classified based on residual GAA activity as CRIM positive patients who have >1% GAA activity or CRIM negative if the residual GAA activity is <1%. In the case of LOPD, cardiomyopathy is quite rare but it causes significant morbidity and mortality in juveniles and adults primarily due to poor lung capacity and functional endurance [1,2].

Enzyme replacement therapy (ERT) is the standard of care treatment for Pompe disease, aiming to restore intracellular levels of GAA. However, the long-term efficacy of ERT is sometimes limited by an immunologic response to repeated doses of rh-GAA [3,4]. In cases of CRIM negative IOPD patients, even a single dose of rh-GAA can elicit a strong immune response abrogating clinical effects. In addition, exogenously added GAA may not reach optimal intracellular concentrations [5]. Gene therapy is a promising modality for an improved therapeutic strategy. To assist in the development of gene therapy for Pompe disease, we sought to identify a biomarker(s) that could provide an easily measurable marker of treatment efficacy or disease progression. Several biomarkers have been utilized to understand the disease progression and monitor the treatment response such as creatine kinase and a tetrasaccharide of glucose (Glc4) which is a by-product of intravascular degradation of glycogen [6,7]. However, creatine kinase is not specific for Pompe disease and is a general marker of muscle injury and Glc4, although quite specific, paints only a picture of intracellular glycogen accumulation in skeletal muscles. Thus, there is an acute need for a specific marker which can reflect the integrity of muscle tissue and structural damage as a result of glycogen accumulation due to the disease progression.

In recent years, there has been considerable interest in exosomes as a source of biomarkers. Exosomes, nano-vesicles released from viable cells, were initially assumed to be a means of cellular waste disposal but there is increasing evidence that they may play a role in intercellular communication [8]. Among the possible signaling molecules found in exosomes are proteins and miRNA. Because exosomes can be harvested from serum, urine, and other biological fluids, we considered them a promising source of biomarkers for Pompe disease. Accordingly, in the present study we compare both protein and miRNA found in exosomes harvested from a Pompe mouse model to healthy littermate controls (LC) and a wild-type strain. In addition, we observed the effect of a candidate gene therapy treatment on potential biomarkers.

## Materials and methods

### Isolation of exosomes

Serum and urine exosomes were isolated using the ExoQuick Exosome precipitation kit (System Biosciences, CA, USA). Briefly, serum and urine were centrifuged at 3000× g for 15 min at 4 °C to eliminate cells and cell debris. The supernatant was transferred to sterile tubes, and an appropriate volume of exosome precipitation solution from the kit was added, with incubation for 30 min at 4 °C. The mixture was then centrifuged at 1500× g for 30 min at 4 °C, and the exosome pellet was re-suspended in sterile phosphate-buffered saline at 4 °C [9,10].

### Proteome analysis

Exosomes were processed for proteomic analysis by Exosome Proteomics Services (System Biosciences, Palo Alto, CA). The protein concentration of each exosome was determined by Qubit fluorometry (Invitrogen, Carlsbad, CA) and each sample (10 µg) was processed by 10% SDS-PAGE. The band was excised, and in-gel digestion was performed using a ProGest robot (DigiLab). The different proteomic contents of Y-exo and O-exo were analyzed using nano liquid chromatography tandem mass spectrometry with a Waters NanoAcquity high-performance liquid chromatography (HPLC) system interfaced to a ThermoFisher Q Exactive. The resulting liquid chromatography tandem mass spectrometry raw data were processed using Sequest as supplied with Proteome Discoverer (Thermo-Fisher) and searched against the UniProt Mouse database. Protein identifications were accepted if a minimum of 2 unique peptides were detected.

Differences in sample size and concentrations were accounted for by quantile normalization, then differences in individual protein abundance were determined by linear regression using R software and the limma package [11]. Differences in protein abundance between groups were considered statistically significant when adjusted P values were below 0.01.

Biological functions enriched in protein sets were identified using the MetaCore program (Clarivate, London UK); the software ranks enriched sets using the hypergeometric distribution algorithm [12]. Heat maps to visualize differences in protein abundance among samples were created with the ComplexHeatMap package in R [13].

### RNA isolation

Total RNA isolation and small RNA purification from serum exosomes were performed using the SeraMir Exosome RNA Purification Column kit. Total RNA isolation and small RNA purification from urine exosomes was performed using the SeraMir Exosome RNA Purification Kit for Media & Urine (SBI Exosome Services, System Biosciences, Palo Alto, CA, USA). For each sample, 1 µL of the final RNA eluate was used for measurement of RNA concentration with the Agilent Bioanalyzer Small RNA Assay using the Bioanalyzer 2100 Expert instrument (Agilent Technologies, Santa Clara, CA, USA).

### RNA sequencing

RNAseq was performed utilizing the Exosome RNA NGS Service from System Biosciences (https://www.systembio.com/services/exosome-services/exo-ngs). Briefly, Illumina NGS libraries were prepared and sequenced using an Illumina HiSeq2000 sequence analyzer (Illumina Inc., San Diego, CA, USA).

### RNA data processing

Raw data were analyzed using the Banana Slug analytics platform (UCSC, CA, USA). Briefly, the exosome Small RNA-seq Analysis kit was initiated with a data quality check of each input sequence using FastQC [14]. Following the quality-control step, the RNA-seq reads were processed to detect and remove spurious nucleotides at the ends of reads, trim sequencing adaptors, and filter reads for quality and length, using FastqMcf, which is part of the EA-utils package (ExpressionAnalysis, NC, USA) and PRINSEQ [15]. FastQC was then repeated to analyze the trimmed reads, thus allowing a ‘before’ and ‘after’ comparison. Sequence reads in the improved set were mapped to the mouse reference genome mm10 using Bowtie [16]. Expression analyses, including computation of read coverage and noncoding RNA abundance, were performed using SAMtools [17]. miRNA abundances were extracted from the list of all small noncoding RNA abundances by filtering for ‘type’ equal to ‘miRNA’ and removing miRNA precursors from the results. Differences in miRNA abundance were calculated using the R programming language with the EdgeR package [18]

### Transgene plasmids

All transgene plasmids contained ITRs sequences derived from AAV2, a synthetic muscle promoter SPc5-12 [19], minute virus of the mouse (MVM) intron [20], WPRE3 [21] and a synthetic polyadenylation sequence [22]. The Dph-CRE04 and the CSk-SH5 transcriptional enhancers were inserted 5′ to the SPc5-12 promoter. Dph-CRE04 is a 509 nucleotide (nt) sequence from the human actin alpha-1 (ACTA1) gene, (SEQ ID NO 4, EP2018/057753). CSk-SH5 is a 454 nt sequence derived from the human filamin C (FLNC) gene (SEQ ID NO 5, EP2015/051081). The transgene consisted of a codon optimized sequence encoding wild type human GAA (hGAA, NCBI accession NP_000143).

### Recombinant AAV production

HEK293 cells were triple transfected with plasmids containing: (1) the AAV2 *Rep* gene and the AAV9 *Cap* gene, (2) a helper plasmid containing adenoviral genes *E2A*, *E4* and *VA*, and (3) the transgene plasmid containing AAV2-derived ITRs. AAV particles were purified either by POROS CaptureSelect AAVX column followed by cesium chloride sedimentation, or by two rounds of cesium chloride sedimentation. Fractions containing mostly full capsids were pooled and subjected to buffer exchange in phosphate buffered saline with 0.001% Pluronic F-68. The genome titer was determined by ddPCR, the purity was assessed by either SDS-PAGE followed by silver staining or CE-SDS to visualize VP1, VP2 and VP3, and the endotoxin was assessed by a lumulus amebocyte lysate assay (Endosafe). Characterized vectors were aliquoted and stored at −80 °C.

### In vivo urine and blood collection

Significant constraints were introduced by the volumes of biological fluid required for exosome isolation from mouse urine and serum. The sole inclusion criterion for the study was genotype (i.e. GAA^−^/GAA^−^ or GAA^+^/GAA^+^). To minimize confounding variables, mice in the control group (GAA^+^/GAA^+^) were healthy littermates of the GAA^−^/GAA^−^ animals. To obtain the volumes of urine required (5–10 ml), urine from 12 to 15 mice (per group) at approximately 5 months of age was manually collected from each mouse daily and pooled (by group) until 5–10 mls was collected for each group. On the final day of the study, 2–3 ml of blood was collected from each mouse by cardiac puncture, pooled by group, and allowed to clot. Serum was then collected following centrifugation. To provide an indication of technical variability, the pools were divided into 2–3 aliquots prior to exosome isolation. For the gene therapy treated groups, 2.5-month-old Pompe (GAA^-^/GAA^-^) mice were given AAV9-GAA and blood and urine was collected when they reached approximately 5 months old. There was no systematic randomization of GAA^−^/GAA^−^ mice for allocation into the treated versus untreated groups. Groups were not blinded at any point during the study. Once the study began, no animals or groups were excluded.

## Results

### Proteomic analysis of EVs

To identify potential protein biomarkers for Pompe disease in exosomes from serum and urine, we performed HPLC-MS/MS on exosomes isolated from mice of three genotypes: GAA^-^/GAA^-^ homozygous knockout, the wild-type strain from which the knockout was derived, and GAA^+^/GAA^+^ littermates of the homozygous knockout mice. The healthy littermates were included as an additional control to account for background genetic drift that may have occurred between the knockout and the ancestral wild-type strain. In addition, the study included a fourth group: GAA^-^/GAA^-^ mice that had been treated with a candidate gene therapy.

### Protein composition of serum exosomes from the GAA knockout is significantly different from wild type

**Figure 1.**
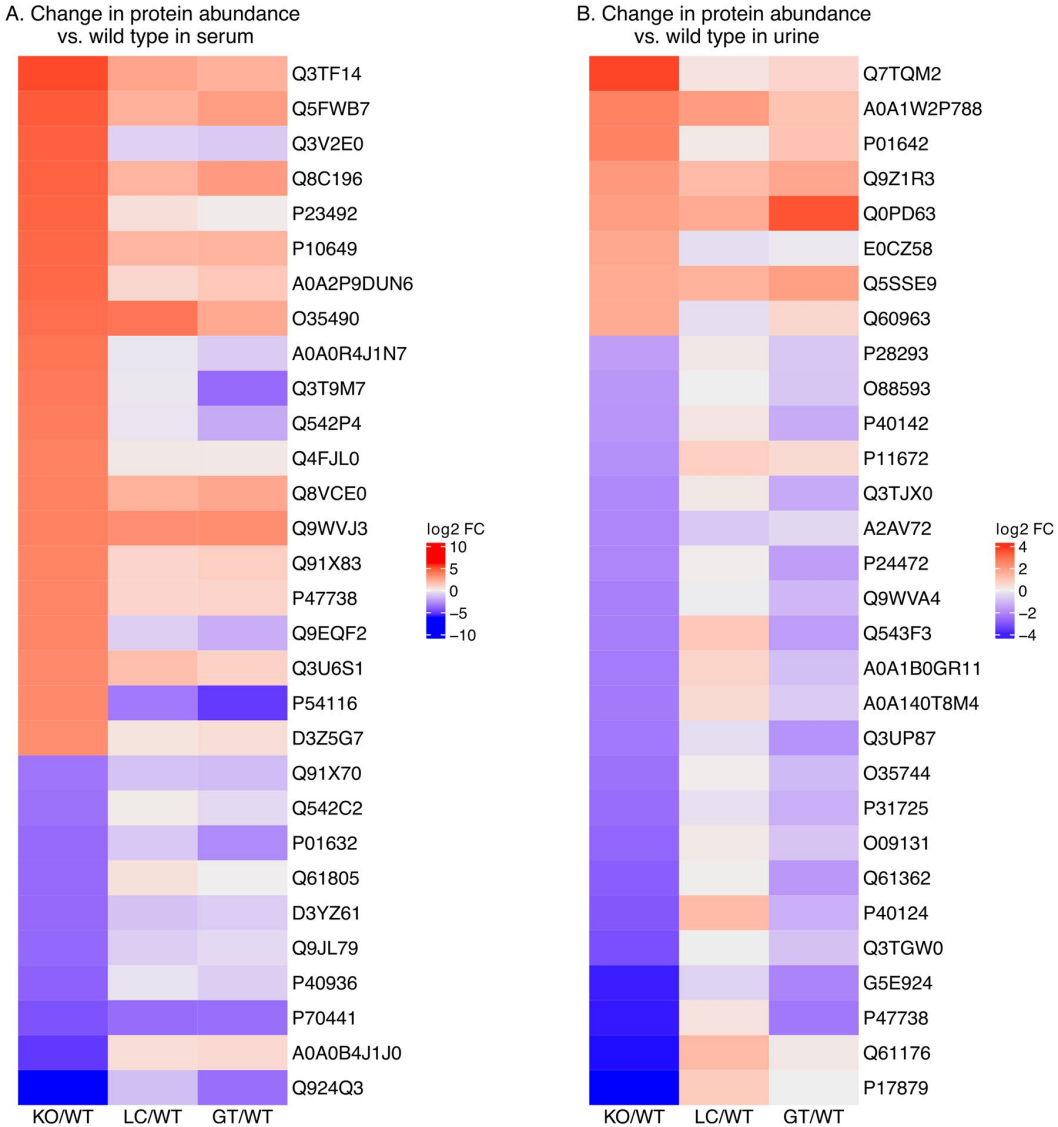
(A) Heat map illustrating difference in abundance of selected proteins in serum exosomes from GAA^−^/GAA^−^, gene therapy treated, and control (LC) mice compared to exosomes of wild-type mice. Proteins selected were those having the greatest difference in abundance between wild-type and GAA^−^/GAA^−^ exosomes. (B) Similar heat map illustrating selected proteins from urine exosomes.

Of 506 proteins detected across all serum samples, 103 were present at significantly higher levels in the untreated GAA^-^/GAA^-^ knockout and 142 were present at significantly lower levels when compared to the ancestral wild-type strain. Figure 1A and Table S1 illustrate the fold change for 30 of these proteins for GAA^-^/GAA^-^ vs. wild type, littermate control vs wild type, and gene therapy treated vs wild type. The 30 proteins shown are those with the greatest change in the knockout compared to the wild type (adjusted *p* value < .01). The heat map illustrates several observations: 1. In general, the greatest differences in abundance are between wild-type and untreated knock-out, 2. There are differences in protein abundance between the wild type and the GAA^+^/GAA^+^ littermates, suggesting that there has been genetic drift, and 3. the levels of serum exosome proteins in the gene-therapy treated knockout mice are very different from the untreated knockout mice, and in fact many protein levels are more similar to the littermate controls, suggesting that the gene therapy treatment may be reducing the effect of the GAA^−^/GAA^−^ phenotype and that this reduction could be monitored *via* serum exosome proteins.

### Protein composition of exosomes isolated from urine

A similar analysis was performed on proteins identified in exosomes isolated from urine from the four groups of mice. The total number of proteins detected in all four groups was similar (556); however, fewer were at significantly different levels when comparing GAA^−^/GAA^−^ to wild type. Eight proteins were more abundant and 24 proteins were less abundant (Table S2). Visualizing the relative abundance of these proteins in GAA^−^/GAA^−^, GAA^+^/GAA^+^, and GAA^−^/GAA^−^ treated mice *via* a heat map suggests similar conclusions as were indicated in the case of serum exosome proteins (Figure 1B).

### Direct comparison of exosomal proteins from GAA^-^/GAA^-^ mice with healthy littermates

The number of generations that have passed since the Pompe model mouse strain branched from the ancestral wild-type strain is unknown. The primary objective of this study was to observe differences between the Pompe mouse model and a GAA^+^ mouse with as similar genetic background as possible. Therefore, in consideration of the differences that we observed in the urine and serum exosome proteomes between the wild-type strain and the littermate controls shown in Figure 1, we performed similar comparisons using littermates as the baseline. There were 131 proteins found at significantly different abundances (Adj.Pval. <.01, FC > 4) between these groups in the exosomes isolated from serum (Table S3). In urine derived exosomes, there were 77 proteins present at different abundances (Table S4).

To test for biological themes in these sets of differentially abundant proteins (in exosomes from GAA^-^/GAA^-^ compared to littermate controls biofluids), we performed pathway enrichment analysis on the protein lists in Tables S3 and S4 using the Metacore platform. These analyses showed that differentially abundant proteins from serum exosomes were frequently involved in lipoprotein metabolism and transport, while differentially abundant proteins from urine exosomes were found more frequently than expected in pathways involved in immune response and cytoskeletal maintenance (Table S5). In both sets of pathway enrichment results, a relatively small set of proteins occurs repeatedly in multiple pathways. Proteins with roles in many pathways frequently interact with many other proteins and therefore are likely to be network hubs. Protein hubs are critical to cellular health and survival [23]. Relative levels of several hub proteins suggested by the pathway enrichment analysis are shown in Tables S6. Note that these proteins are present in reduced quantities in GAA^−^/GAA^−^ exosomes, but that treatment with the AAV9-GAA gene therapy candidate results in an increasing abundance of hub proteins, closer to the LC level.

### miRNA isolated from serum exosomes

**Figure 2.**
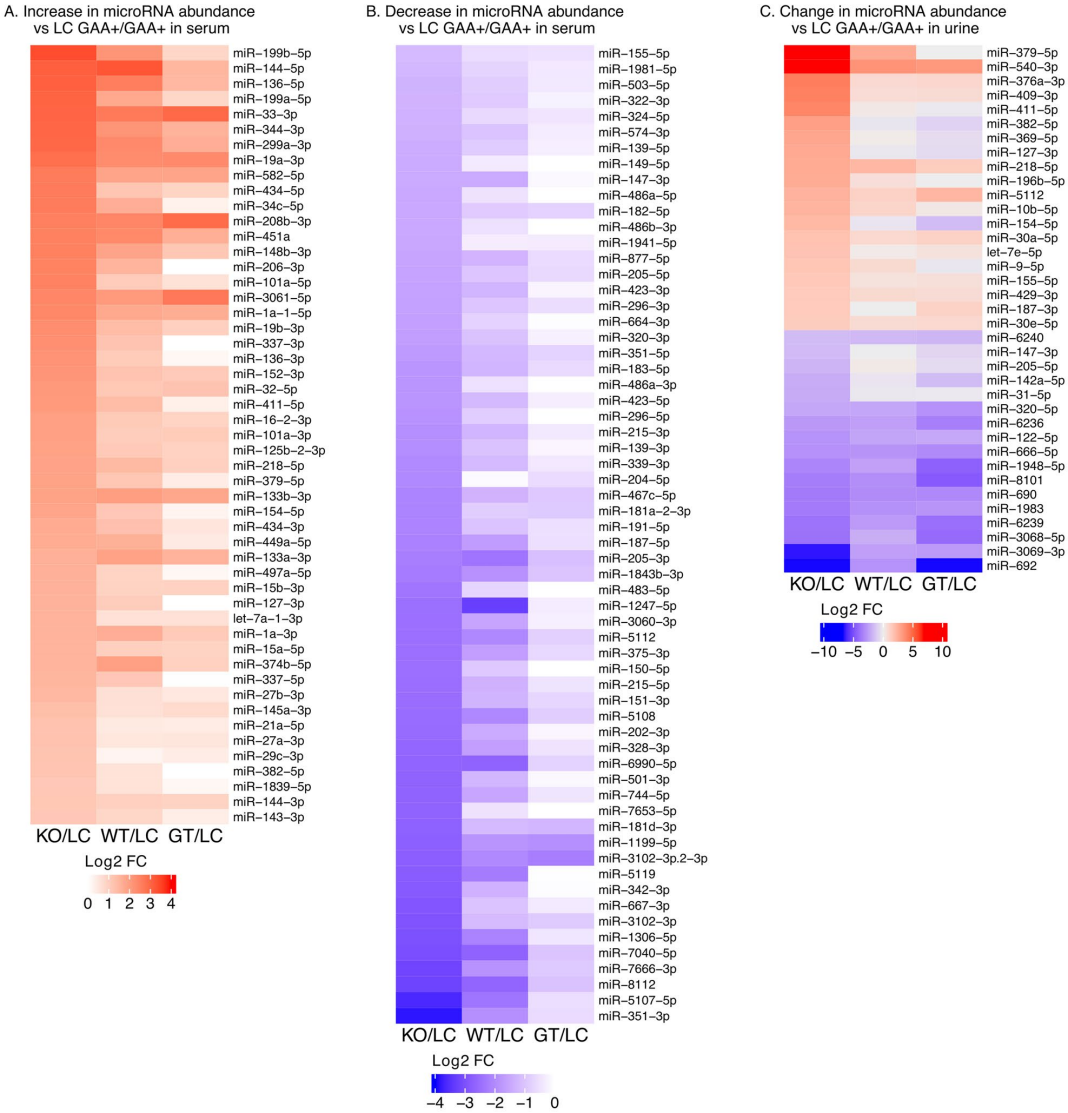
A. Heat map illustrating miRNA fold change (FC) in serum exosomes from GAA^−^/GAA^−^ knock-out (KO), wild-type, and gene therapy-treated KO compared to GAA+/GAA + littermate controls (LC). All miRNA with fold change for KO vs LC of 2 or greater (i.e. log2(FC) > = 1, adjusted Pval < = .05) are shown. B. All miRNA in serum exosomes with fold change for KO vs LC of 0.5 or less. C. All miRNA with fold change for KO vs LC of greater than 2.0 or less than 0.5 (adjusted Pval < = .05) in exosomes from urine.

In addition to exosome proteins, we considered the possibility that exosomal miRNAs may be useful biomarkers of Pompe disease. We isolated and quantified miRNA from exosomes derived from the Pompe mouse model serum and urine and from healthy littermate serum and urine. A total of 472 unique miRNA were quantified from serum exosomes. There were significant (Adj.Pval. < .05, FC > 2) differences between PD and littermate controls in the abundance of 113 of them (Figure 2A,B and Table S7).

The list of differentially abundant miRNA in serum exosomes contains several important regulators of muscle development and maintenance. microRNAs miR-206, miR-208b, miR-133a, miR-133b, and miR-1a are considered myomirs because they are expressed exclusively in muscle. MicroRNA miR-486 is highly enriched in muscle and is also a myomir [24]. Some myomirs, including miR-1a, miR-206, miR-133a, and miR-133b are known as dystromirs because of their altered regulation in Duchenne Muscular Dystrophy (DMD) and other dystrophies [25]. As seen in Table 1, the myomirs detected were present at altered levels in serum exosomes of the Pompe model when compared to littermate controls. The AAV9-GAA treatment restored or partially restored the miRNA level to that of the littermate controls for several myomirs (e.g. miR-206, miR-486 family). Other miRNAs were affected to a lesser extent, or not at all, by the gene therapy (e.g. miR-133, miR-1a).

**Table 1.** Myomirs (miRNA expressed exclusively or highly enriched in muscle) differentially abundant in exosomes from Pompe model serum compared to littermate control serum.

Differentially abundant myomirs in PD mouse serum exosomes			
miRNA_ID	FDR	Fold Change KO vs LC	Fold Change GT vs LC
miR-208b-3p	1.13E-02	5.93	7.60
miR-206-3p	2.86E-18	5.82	0.95
miR-133b-3p	8.23E-05	3.75	3.52
miR-133a-3p	3.47E-09	3.14	2.97
miR-1a-3p	1.04E-08	2.98	2.17
miR-486a-5p	3.20E-04	0.36	1.31
miR-486b-3p	4.15E-03	0.36	1.22
miR-486a-3p	2.03E-03	0.28	1.20

In addition to the known myomirs, there are broadly expressed microRNA that have regulatory functions in muscle proliferation, differentiation, and maintenance. Table S8 lists 19 microRNA, including myomirs and more widely expressed miRNA, that were dysregulated in serum exososmes of GAA-/GAA- mice compared to littermate controls. Tarallo et al. [26] examined the miRNA content of Pompe mouse heart and gastrocnemii at age three and nine months. In our study of serum exosomes, we observed many muscle-specific miRNA; therefore, it is likely that some of the serum exosome fraction originated from muscle tissue. It was of interest to see the extent of overlap between miRNA dysregulated in both studies. As shown in Table S8, of the 19 muscle-related miRNAs present in altered amounts in serum exosomes from PD mice, 14 were also observed to be expressed at altered levels in heart and/or gastrocnemius from PD mice. To obtain a more comprehensive view of the commonality between miRNA transcriptomes obtained *via* these two sources, we compared the 113 miRNAs found at different abundances in the present study to 97 miRNAs that were differentially expressed in both gastrocnemius and heart at both three and nine months. As shown in Table S9, 24 mature miRNAs were seen in both studies; thus, approximately twenty percent of the miRNA seen in each study is in common with the other. We noted also that there are five miRNAs in the present study that were not directly observed in the gastrocnemius/heart study; however, the miRNA from the opposite arm of the precursor miRNA was seen.

### miRNA isolated from urine exosomes

A total of 188 unique miRNAs were detected in urine exosomes from PD mice and littermate controls. There were significant differences in the abundance of 37 of these in PD mice compared to littermate controls (Figure 2C, Table S10). Twenty microRNA were significantly more abundant in PD, and 17 microRNA were present in lower amounts. Unlike the exosome fraction from serum, exosomes from urine did not contain an abundance of myomirs or dystromirs.

However, the six miRNAs from PD urine with the highest fold change compared to controls (Table 2) are all located in the Dlk1-Dio3 locus of mouse chromosome 12. Five of the six (miRNA-540 is the exception) are part of the miR-379 microRNA megacluster. These and other members of the cluster are coordinately expressed and are involved in the regulation of muscle development [27].

**Table 2.** miRNAs with greatest fold change in urine exosomes in knockout compared to littermate controls.

Most differentially abundant miRNA in PD mouse urine exosomes			
mRNA_ID	FDR	Fold Change KO vs LC	Fold Change GT vs LC
miR-379-5p	1.04E-04	152	1.00
miR-540-3p	2.18E-04	115	10.21
miR-376a-3p	2.21E-05	20	1.93
miR-409-3p	1.19E-04	17	1.69
miR-411-5p	1.32E-09	16	0.90
miR-382-5p	1.10E-08	8.5	0.54

### Relationship between miRNA and protein changes in serum exosomes from PD mice

miRNAs negatively regulate gene expression by binding to target areas in the 3′ end of messenger RNAs and promoting degradation or inhibiting translation [28,29]. Therefore, it was of interest to investigate whether a relationship could be detected between miRNA and proteins that are significantly changed in EV from Pompe mice. Using the TargetScan database [28], we collected 2961 genes that have predicted target sites for three or more of the 51 miRNA that are significantly upregulated in PD mice. We then searched this list for genes that encode proteins that are significantly altered in exosomes from PD mice (Table 3). The final list contains six of the 131 proteins that were present in altered amounts. Interestingly, all six of the proteins are present in increased amounts in the PD animals and their levels are reduced by the candidate gene therapy treatment. miRNAs are negative regulators; therefore, the increase in abundance of these proteins in PD is likely a result of another regulatory circuit(s) – the observed increase in miRNAs may be a response rather than a cause of the increased target abundance. The ankyrin protein is a particularly interesting case; it has been shown to have an important role in stabilizing the structure of muscle fibers [30]. Ankyrin was markedly increased in PD mice (FC 19.4, adjusted Pval .002). The corresponding ankyrin mRNA contains potential target sites for miR-27a-3p, miR-27b-3p, miR-582-5p, and miR-29c-3p, microRNA that are present at increased levels in PD mice. The ankyrin gene, Ank1, encodes miR-486 in addition to the ankyrin protein. In the present study, in contrast to the upregulation of ankyrin, miRNAs in the mir-486 family were down-regulated with fold change 3-4X. While these observations appear contradictory, it is important to consider that miRNA are subject to considerable post-transcriptional regulation [31]. The multiple interconnections between an important muscle structural protein and several miRNAs emphasizes the complex regulatory network that is perturbed in Pompe Disease.

**Table 3.** Proteins present at altered levels in serum exosomes of PD mice that are encoded by genes with target sites for three or more miRNA dysregulated in PD serum exosomes.

		Protein Fold Change			Predicted binding sites on mRNA for miRNA upregulated in serum exosomes
Protein Accession	Description	KOvsLC	GTvsLC	WTvsLC	
A0A0R4J1N7	Ankyrin-1	19.4	0.62	1.24	miR-27a-3p, miR-27b-3p, miR-582-5p, miR-29c-3p
D1FNM9	Calcium-transporting ATPase	7.27	1.02	0.92	miR-34c-5p, miR-449a-5p, miR-411-5p, miR-29c-3p, miR-449a-5p, miR-34c-5p, miR-152-3p, miR-148b-3p
E9PY39	Predicted gene 20431	71.35	48.42	53.69	miR-27a-3p, miR-27b-3p, miR-15a-5p, miR-497a-5p
Q4FJL0	Rab10	11.04	1.02	1.03	miR-374b-5p, miR-497a-5p, miR-15a-5p, miR-199a-5p, miR-411-5p
P61028	Ras-related protein Rab-8B	5.32	1.63	1.62	miR-218-5p, miR-19a-3p, miR-19b-3p, miR-32-5p
Q52L50	RAS related protein 1b	16.58	2.23	2.93	miR-1a-3p, miR-206-3p, miR-19a-3p, miR-19b-3p, miR-144-3p, miR-374b-5p, miR-27b-3p, miR-27a-3p, miR-32-5p

### Effect of AAV9-GAA gene therapy on lysosomal alpha-glucosidease and glycogen levels in tissues

**Figure 3.**
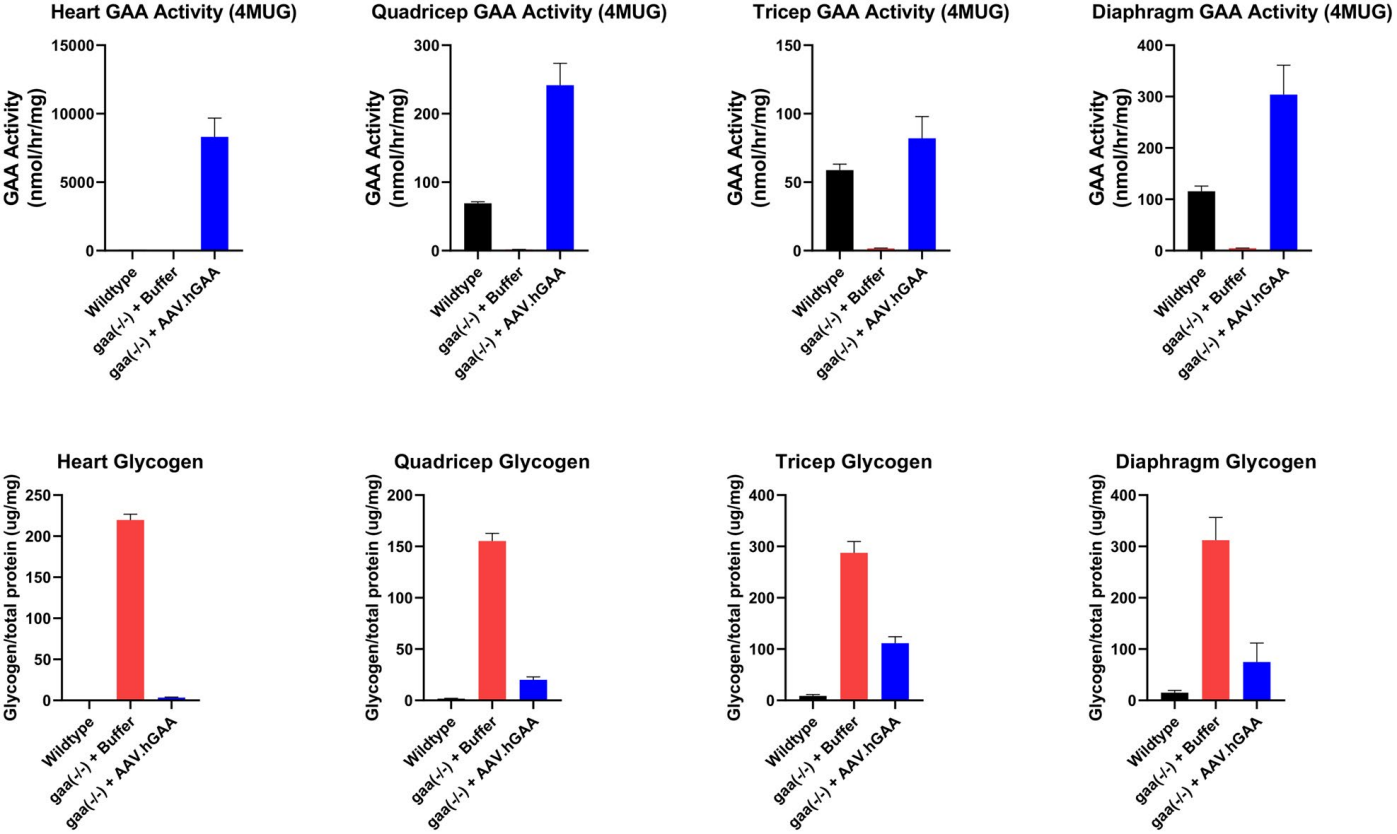
AAV delivery of a GAA transgene to Pompe mice exhibited increased GAA activity and a decrease in glycogen in all tissues examined compared to untreated and WT mice. 2.5-month-old mice were dosed with 1e14 vg/kg of AAV9-GAA systemically. Tissues were collected when the mice reached 5 months of age.

Systemic administration of 1e14 vg/kg of AAV9-GAA resulted in GAA activity levels in tissue homogenates that exceeded WT GAA activity levels in all assessed tissues (heart, quadricep, tricep, diaphragm). A greater than 75% reduction in glycogen was seen in the heart, quadricep, and diaphragm tissues. A modest 60% reduction in glycogen was observed in tricep (Figure 3).

## Discussion

In this study, we profile both the miRNA and protein content of exosomes from serum and urine from a Pompe Disease mouse model. We observed extensive changes in the abundance of specific miRNAs and proteins. The primary motivation for the study was to identify PD biomarkers from non-invasive sources, for the purpose of monitoring response to candidate gene therapy treatments.

Numerous studies have highlighted the potential for circulating myomirs (muscle specific or muscle enriched miRNAs) to serve as biomarkers for muscle damage or disease (for review, see Ref. [29]). miRNAs miR-206, miR-133a and miR-1a-3p were found to be present at increased levels in DMD and other dystrophies and have been termed dystromirs [32,33]. In Pompe Disease, Carrasco-Rozas et al. [34], identified 14 miRNA, including the three dystromirs, that were present in altered amounts in serum from AOPD patients. Tarallo et al. [26] measured miRNA levels in cardiac and gastrocnemius muscle in the mouse PD model, and in plasma from Pompe patients. In the study presented here, we identified changes in both miRNA and proteins found in exosomes from serum and urine. One distinction gained by limiting analysis to the exosome fraction of these biofluids derives from the fact that exosomes are produced *via* a specific cellular process, which is initiated at the endocytic cisterna, then involves the accumulation of specific cargoes, and finally release into the extracellular space and circulation where the exosomes migrate and interact with target cells, thereby effecting intercellular communication [35]. From a technical perspective, an additional advantage to studying proteins from serum exosomes compared to whole serum is that exosomal proteins represent a purified fraction, with less interference by major serum proteins such as albumin [36,37].

In the present study, we observed extensive changes in both the protein and miRNA content of exosomes derived from serum and urine from PD mice. However, before considering the miRNA data, analysis of the differences between exosomal proteomes of PD and control mice did not reveal a clear link to previously recognized characteristics of Pompe disease or to other myopathies.

In contrast, the miRNAs found in exosomes from PD mice provide a strong indication that there are alterations in muscle tissue. In urine, we observed an abundance of miRNA encoded within the Dlk1-Dio3 locus, which contains important regulators of muscle growth. For example, the abundance of miR-540-3p was increased 115-fold in PD mice; this miRNA has been shown to increase myotube diameter in *in vitro* transfection experiments. An additional five miRNA that we observed to be increased in PD are located on the miR-379 megacluster, which also maps to this region. Transcription of the miR-379 megacluster and miR-540-3p is reduced by myostatin, a transforming growth factor-beta family cytokine that inhibits muscle development [27]. Altered levels of several of miRNAs from this region have previously been observed in PD mouse heart and gastrocnemius tissue [26]. Overall, these data indicate that it may be possible to detect the perturbations in muscle regulatory circuitry in Pompe Disease by monitoring miRNA in urine exosomes.

In serum exosomes, we observed altered levels of 113 microRNA in PD mice. At least 19 of these are known to be involved in the regulation of muscle development or maintenance. Several of these (miR-208b-3p, miR-206-3p, miR-133a-3p, miR-133b-3p, miR-1a-3p, and members of the miR-486 family) are known as myomirs because they are exclusive to or enriched in muscle tissue [24]. In the present study, all myomirs except those in the miR-486 family were observed at higher levels in serum EVs of PD mice vs. healthy mice (Fold Change ranged from 2.98 to 5.93). In contrast, the miR-486 family miRNAs were observed at lower levels in PD mice. A similar expression profile is seen in DMD [38].

Myomirs and other miRNAs regulate different skeletal muscle-related processes such as muscle regeneration/degeneration, growth, atrophy, etc. Some of these miRNAs, being selectively expressed by heart and skeletal muscle, provide a glimpse of underlying disease-related muscle pathology. miRNA-133 plays a role in the proliferation of myoblasts and miRNA-206 is involved in the differentiation of myoblasts to myotubes. Experiments with knock-out mouse models have demonstrated that miR-486 is required for normal skeletal and cardiac muscle growth [38]. As many of these miRNAs play a part in muscle regeneration, it is reasonable to expect their differential expression in situations where the muscle degenerative process is active. Additionally, miRNA-206 and 133a have been shown to be significantly elevated in symptomatic LOPD and IOPD patients, signifying their utility for prognostic purposes. Since ERT, the current standard of care treatment, does not address the underlying genetic pathophysiology of the disease, it does not significantly reduce or normalize the elevated levels of these miRNAs in treated patients. Indeed, this has been shown recently in a study involving adult-onset LOPD patients where the ERT therapy for 6 months to 1 year failed to normalize the serum levels of dystromirs in most patients [34]. On the other hand, gene therapy, with its ability to address the underlying root-cause of the disease, would be expected to significantly normalize the levels of such muscle-specific miRNAs. With AAV9-GAA treatment given at 2.5 months of age to Pompe mice, we have shown the restoration of the levels of miR-206 and miR-486a/b to control level at 5 months of age. During this period, miR-133a/b, miR-208-3p, and miR-1a-3p were not significantly impacted. The mechanism underlying the varying response of different miRNAs is not clear. In the case of miR-133a this may be due the fact that miR-133a is only modestly elevated in Pompe mouse model at 15 months of age and represents a very mild phenotype [26]. The effects of gene therapy are expected to ramp up and show even higher significance as these treated mice age further. Thus, these miRNAs can not only be used to assess the treatment response but could also indicate potential differentiation with respect to the standard of care. Moreover, in addition to their possible clinical importance, further study of miRNA in exosomes from Pompe animal models may yield new insights into the mechanisms of disease.

## Supplementary Material

Supplemental Material

## Data Availability

The data that support the findings of this study will be openly available in Zenodo at http://doi.org/10.5281/zenodo.8301798 upon publication.
