# Supplementary material for: Combined miRNA transcriptome and proteome analysis of extracellular vesicles in urine and blood from the Pompe mouse model
Source: Ann Med. 2024 Oct 24;56(1):2402503. doi: 10.1080/07853890.2024.2402503 (PMC11504521; doi:10.1080/07853890.2024.2402503)
Supplement: Supplemental Material [file IANN_A_2402503_SM4827.zip › Suppl_Mat tables/SupplementaryTableLegends_Final.docx]

Supplementary Table Legends

Table S1 – Comparison of abundance of selected proteins in serum exosomes from GAA-/GAA-, gene therapy treated, and littermate control mice compared to exosomes of wild-type mice. Proteins selected were those having the greatest difference in abundance between wild-type and GAA-/GAA- exosomes.

Table S2 - Comparison of abundance of proteins in urine exosomes from GAA-/GAA-, gene therapy treated, and littermate control mice compared to exosomes of wild-type mice. Proteins selected were all those significantly different in abundance between wild-type and GAA-/GAA- exosomes.

Table S3 – Comparison of abundance of proteins in serum exosomes from wild-type, GAA-/GAA, and gene therapy treated mice, compared to exosomes of littermate control mice. Proteins selected were all those significantly different in abundance between control and GAA-/GAA- exosomes.

Table S4 – Comparison of abundance of proteins in urine exosomes from wild-type, GAA-/GAA-, and gene therapy treated mice, compared to exosomes of littermate control mice. Proteins selected were all those significantly different in abundance between control and GAA-/GAA- exosomes.

Table S5 – Pathways enriched for proteins differentially abundant in exosomes from GAA-/GAA- mice compared to littermate control mice.

Table S6 – Proteins common to four or more pathways enriched for differentially abundant exosome proteins (A. GAA-/GAA- serum exosomes compared to GAA+/GAA+ serum exosomes, B. GAA-/GAA- urine exosomes compared to GAA+/GAA+ urine exosomes).

Table S7 - Comparison of abundance of miRNA in serum exosomes from knock-out, gene therapy treated knock-out, and wild type compared to exosomes of heterozygous mice. MiRNA selected were all those significantly different in abundance between littermate control and GAA-/GAA- exosomes.

Table S8 – miRNA observed at altered levels in PD mouse serum exosomes that are known to regulate muscle development, differentiation, or maintenance. Also shown (column E) is presence/absence of miRNA in mouse gastrocnemius or heart (from Figure 1 in Reference 26). Below are references for role in muscle development and maintenance (columns F and G; references 25-34 also appear in main text, 39-52 appear only in Table S8):

1. Zaharieva IT, Calissano M, Scoto M, et al. Dystromirs as serum biomarkers for monitoring the disease severity in Duchenne muscular dystrophy. PLOS One. 2013;8(11):e80263. doi: 10.1371/journal.pone.0080263.
2. Tarallo A, Carissimo A, Gatto F, et al. MicroRNAs as biomarkers in Pompe disease. Genet Med. 2019;21(3):591–600. doi: 10.1038/s41436-018-0103-8.
3. Hitachi K, Tsuchida K. Myostatin-deficiency in mice increases global gene expression at the Dlk1-Dio3 locus in the skeletal muscle. Oncotarget. 2017;8(4):5943–5953. doi: 10.18632/oncotarget.13966
4. Siracusa J, Koulmann N, Banzet S. Circulating myomiRs: a new class of biomarkers to monitor skeletal muscle in physiology and medicine. J Cachexia Sarcopenia Muscle. 2018;9(1):20–27. doi: 10.1002/jcsm.12227.
5. Carrasco-Rozas A, Fernández-Simón E, Lleixà MC, et al. Identification of serum microRNAs as potential biomarkers in Pompe Disease. Ann Clin Transl Neurol. 2019;6(7):1214–1224. doi: 10.1002/acn3.50800.
6. Javed R, Jing L, Yang J, et al. miRNA transcriptome of hypertrophic skeletal muscle with overexpressed myostatin propeptide. Biomed Res Int. 2014;2014:e328935–19. doi: 10.1155/2014/328935.
7. Zhai L, Wu R, Han W, et al. miR-127 enhances myogenic cell differentiation by targeting S1PR3. Cell Death Dis. 2017;8(3):e2707. doi: 10.1038/cddis.2017.128.
8. Chen J-F, Mandel EM, Thomson JM, et al. The role of microRNA-1 and microRNA-133 in skeletal muscle proliferation and differentiation. Nat Genet. 2006;38(2):228–233. doi: 10.1038/ng1725.
9. Soriano-Arroquia A, House L, Tregilgas L, et al. The functional consequences of age-related changes in microRNA expression in skeletal muscle. Biogerontology. 2016;17(3):641–654. doi: 10.1007/s10522-016-9638-8.
10. Li N, Hwangbo C, Jaba IM, et al. miR-182 modulates myocardial hypertrophic response induced by angiogenesis in heart. Sci Rep. 2016;6(1):21228. doi: 10.1038/srep21228.
11. Berardi E, Annibali D, Cassano M, et al. Molecular and cell-based therapies for muscle degenerations: a road under construction. Front Physiol. 2014;5:119. doi: 10.3389/fphys.2014.00119.
12. Van Rooij E, Liu N, Olson EN. MicroRNAs flex their muscles. Trends Genet. 2008;24(4):159–166. doi: 10.1016/j.tig.2008.01.007.
13. Van Rooij E, Quiat D, Johnson BA, et al. A family of ­microRNAs encoded by myosin genes governs myosin expression and muscle performance. Dev Cell. 2009;17(5):662–673. doi: 10.1016/j.devcel.2009.10.013.
14. Haider KH, Idris NM, Kim HW, et al. MicroRNA-21 is a key determinant in IL-11/Stat3 anti-apoptotic signalling pathway in preconditioning of skeletal myoblasts. Cardiovasc Res. 2010;88(1):168–178. doi: 10.1093/cvr/cvq151.
15. Crist CG, Montarras D, Pallafacchina G, et al. Muscle stem cell behavior is modified by microRNA-27 regulation of Pax3 expression. Proc Natl Acad Sci U S A. 2009;106(32):13383–13387. doi: 10.1073/pnas.0900210106.
16. McFarlane C, Vajjala A, Arigela H, et al. Negative auto-regulation of myostatin expression is mediated by Smad3 and MicroRNA-27. PLOS One. 2014;9(1):e87687. doi: 10.1371/journal.pone.0087687.
17. Zhang Y, Liu C, Wang J, et al. MiR-299-5p regulates apoptosis through autophagy in neurons and ameliorates cognitive capacity in APPswe/PS1dE9 mice. Sci Rep. 2016;6(1):24566. doi: 10.1038/srep24566.
18. Hou L, Xu J, Li H, et al. MiR-34c represses muscle development by forming a regulatory loop with Notch1. Sci Rep. 2017;7(1):9346. doi: 10.1038/s41598-017-09688-y.
19. Ghafouri-Fard S, Shaterabadi D, Abak A, et al. An update on the role of miR-379 in human disorders. Biomed Pharmacother. 2021;139:111553. doi: 10.1016/j.biopha.2021.111553.

Table S9 – Comparison of dysregulated miRNA observed in serum exosomes from PD mice in the present study with miRNA altered in skeletal or cardiac muscle in PD mice at 3 months and 9 months of age (from supplementary data, Tarallo et al. 2019).

Table S10 – Comparison of abundance of miRNA in urine exosomes from knock-out, gene therapy treated knock-out, and wild type compared to exosomes of littermate control mice. MiRNA selected were all those significantly different in abundance between control and GAA-/GAA- exosomes.
